# Drug–drug interactions between treatment specific pharmacotherapy and concomitant medication in patients with COVID-19 in the first wave in Spain

**DOI:** 10.1038/s41598-021-91953-2

**Published:** 2021-06-14

**Authors:** M. D. Cantudo-Cuenca, Antonio Gutiérrez-Pizarraya, Ana Pinilla-Fernández, Enrique Contreras-Macías, M. Fernández‑Fuertes, F. A Lao‑Domínguez, Pilar Rincón, Juan Antonio Pineda, Juan Macías, Ramón Morillo-Verdugo

**Affiliations:** 1 Pharmacy Unit, Hospital Universitario Virgen de Valme, Ctra. de Cádiz Km. 548,9, Seville, Spain; 2 Infectious Diseases and Microbiology Unit, Hospital Universitario Virgen de Valme, Avda. Bellavista s/n, 41014 Seville, Spain

**Keywords:** Infectious diseases, Adverse effects, Drug therapy

## Abstract

Primary aim was to assess prevalence and severity of potential and real drug–drug interactions (DDIs) among therapies for COVID-19 and concomitant medications in hospitalized patients with confirmed SARS-CoV-2 infection. The secondary aim was to analyze factors associated with rDDIs. An observational single center cohort study conducted at a tertiary hospital in Spain from March 1st to April 30th. rDDIs refer to interaction with concomitant drugs prescribed during hospital stay whereas potential DDIs (pDDIs) refer to those with domiciliary medication. DDIs checked with The University of Liverpool resource. Concomitant medications were categorized according to the Anatomical Therapeutic Chemical classification system. Binomial logistic regression was carried out to identify factors associated with rDDIs. A total of 174 patients were analyzed. DDIs were detected in 152 patients (87.4%) with a total of 417 rDDIs between COVID19-related drugs and involved hospital concomitant medication (60 different drugs) while pDDIs were detected in 105 patients (72.9%) with a total of 553 pDDIs. From all 417 rDDIs, 43.2% (n = 180) were associated with lopinavir/ritonavir and 52.9% (n = 221) with hydroxychloroquine, both of them the most prescribed (106 and 165 patients, respectively). The main mechanism of interaction observed was QTc prolongation. Clinically relevant rDDIs were identified among 81.1% (n = 338) (‘potential interactions’) and 14.6% (n = 61) (contraindicated) of the patients. Charlson index (OR 1.34, 95% IC 1.02–1.76) and number of drugs prescribed during admission (OR 1.42, 95% IC 1.12–1.81) were independently associated with rDDIs. Prevalence of patients with real and pDDIs was high, especially those clinically relevant. Both comorbidities and polypharmacy were found as risk factors independently associated with DDIs development.

## Introduction

In December 2019, China reported a cluster of pneumonia cases of unknown cause that would later be identified as severe acute respiratory syndrome coronavirus 2 (SARS-CoV-2)^1^. As of January 26, 2021, confirmed cases have risen to more than 100,000,000 worldwide and more than 2,144,000 people have died. Spain has one of the highest 2019 coronavirus diseases (COVID-19) clinical burdens in the world^2^, nowadays, more two million infections have been reached.

COVID-19 presents varied clinical features, ranging from asymptomatic to acute respiratory distress syndrome (ARDS)^3,4^. The most common clinical manifestations are fever, cough, shortness of breath, myalgia or fatigue. Headache, confusion, rhinorrhoea, haemoptysis, vomiting, and diarrhoea have been reported but are less common^3–6^. Anosmia or ageusia preceding the onset of respiratory symptoms has been anecdotally reported^7^. The incubation period for COVID-19 is thought to extend to 14 days, with a median time of 4–5 days from exposure to symptoms onset^8,9^. Clinical management includes infection control measures and supportive care, including supplemental oxygen and mechanical ventilation when indicated^10^. There is currently no approved specific treatment that improves clinical outcomes in patients with either suspected or confirmed COVID-19^11^. So far, only remdesivir and dexamethasone have shown some usefulness. In the first case, in a randomized study compared to placebo in which remdesivir showed a reduction in the median time to recovery, being 15 days for the placebo group and 11 days for the treated group with remdesivir^12^. For the case of dexamethasone, in a preliminary report of the RECOVERY clinical trial^13^ in patients hospitalized with COVID-19, the use of this drug has resulted in lower 28-day mortality among those who were receiving either invasive mechanical ventilation or oxygen alone at randomization but not among those receiving no respiratory support. In this sense, WHO SOLIDARITY trial has found no benefit for any of the above drugs^14^. In addition, very recently, an international, randomized, placebo-controlled collaborative trial, REMAP- CAP has published positive results for tocilizumab/sarilumab in ICU patients if administered very soon after admission^15^.

Antimicrobial agents with potential activity against SARS-CoV-2 used in Spain during the first coronavirus wave were: remdesivir, hydroxychloroquine, lopinavir–ritonavir, azithromycin and the adjunctive therapies including, interferon beta1-B (IFNb), systemic corticosteroids, tocilizumab, immunoglobulin and anakinra^16^. The health emergency caused by COVID-19 pandemic and the use of therapeutic agents with high uncertainty and/or potential for harm, based on limited available evidence. The exponential increase in hospital admissions and the age range of the patients, could have led to high rates of drug-drug interactions (DDI), specially in the first wave of the pandemic with the drugs that were used at that time. Later, from June to September 2020, the pharmacological management changed due to the knowledge that emerged from the epidemiological trials and studies carried out with data from the first wave. That interactions may constitute one of the potential mechanisms of preventable adverse drug events and health damage^17^. COVID-19 therapies can contribute to a host of DDIs. Pharmacokinetic interactions, induction/inhibition of cytochrome isoforms, and pharmacodynamic DDIs can also be relevant, in particular because hydroxychloroquine, lopinavir/ritonavir and macrolide agents can cause prolonged QT and Torsade de Pointes (TdP)^18^. This risk could be further amplified if multiple medications, each with their own QTc-prolonging and torsadogenic potential, are used in combination^19,20^. Moreover, the presence of comorbidities such as hypertension, diabetes, obesity, coronary heart disease and chronic obstructive lung disease are frequent, and they may also receive multiple medications, causing adverse effects or DDIs^21,22^. Due to all of the above, careful review of concomitant medications and monitoring are required if this drug is used.

Currently, limited data is available about potential and real risk prevalence in clinical practice of these drugs, associated with patterns of drug use, so it is necessary to provide data to further improve this key aspect for optimal patient management. Therefore, this study aims to assess prevalence of DDIs among experimental drugs for COVID-19 and concomitant medications in patients needing hospital admission. We also categorized DDIs according to interaction risk and severity. Finally, we analysed factors associated with real DDIs.

## Results

### Baseline features

A total of 205 patients were included. Thirty-one patients were excluded from the analysis because they did not meet inclusion criteria; 28 patients of them had not received specific treatment for COVID-19, two were under 18 years old and one was discharged before the first 24 h. Thus, we analysed a total of 174 patients, 88 (50.6%) were male, with a median age of 67 years (IQR 54–73) The rest of demographic and clinical characteristics are shown in Table 1.

**Table 1:** Primary diagnoses, underlying diseases, and clinical characteristics of the patients.

	Total cohort N = 174 (%)
Median age (IQR) (yr)	67 (54–73)
Gender (male)	88 (50.6)
Charlson index (IQR)	4 (2–6)
**Underlying diseases**	
**Hypertension**	84 (48.3)
Myocardial infarction	23 (13.4)
Cerebrovascular accident	22 (12.8)
Diabetes mellitus	45 (26.2)
Dementia	37 (21.5)
COPD^a^	10 (5.8)
Asthma	10 (5.8)
Chronic renal insufficiency	18 (10.5)
Cancer	19 (11)
**COVID-19 pharmacotherapy**	
Lopinavir/ritonavir	106 (62.4)
Hydroxychloroquine	165 (97.1)
Azithromycin	36 (21.3)
Tocilizumab	13 (7.6)
Metilprednisolone	61 (35.9)
Anakinra	6 (3.5)
**Hospital no Covid pharmacotherapy**	
ACEIs^b^	34 (23.4)
ARBs^c^	28 (19.4)
BBs^d^	32 (21.9)
Statins	25 (14.4)
**LMWH**^**e**^	
Prophylactic	131 (86.8)
Treatment	44 (42.7)
ICU admission	16 (9.2)
APACHE II^f^	15 (14–16)
SOFA^g^	6 (5–4)
Hospital stays	11 (7–17)
Hospital mortality	40 (23.5)

^a^COPD denotes Chronic obstructive pulmonary disease.

^b^Angiotensin-converting enzyme inhibitors.

^c^Angiotensin receptor blockers.

^d^Beta blockers.

^e^Low-molecular-weight heparin.

^f^APACHE II denotes Acute Physiology and Chronic Health Evaluation score.

^g^SOFA denotes Sequential Organ Failure Assessment.

The most common comorbidities were hypertension (n = 84; 48.3%) and diabetes (n = 45; 26.2%). The median hospital stay was 11 (6–17) days. During hospitalization, 16 patients (9.2%) were admitted to the ICU, 7 of which (43.7%) received invasive mechanical ventilation. Overall, 40 patients (22.9%) died during hospital stay.

The most frequently used drugs were hydroxychloroquine (n = 165, 94.8%) and lopinavir/ritonavir (n = 106, 60.9%). Antibiotics were indicated, mainly beta-lactam antibiotics (n = 66, 37.9%), levofloxacin (n = 7, 4.1%) and azithromycin (n = 36, 20.7%). Immunomodulatory drugs were also common, such as corticosteroids (n = 62, 35.6%), interferon beta 1-B (n = 13, 7.5%), tocilizumab (n = 13, 7.5%), immunoglobulin (n = 2, 1.1%) and anakinra (n = 6, 3.4%).

### DDI prevalence and severity

DDIs were detected in 152 patients (87.4%) with a total of 417 interactions between COVID19-related drugs and other concomitant medications used during hospital stay. Of these, 43.2% (n = 180) were associated with lopinavir/ritonavir and 52.9% (n = 221) with hydroxychloroquine. The most implicated drugs were 10.6% amlodipine (n = 19) followed by 8.3% furosemide (n = 15) and 13.6% furosemide (n = 30) and 11.8% insulin (n = 26), respectively. According to clinically relevant DDIs, 81.1% were “potential interaction” (n = 338) and 14.6% contraindicated (n = 61). Hydroxychloroquine was the most common drug involved in amber (n = 187, 55.3%) and red DDIs (n = 31, 53.4%) followed by lopinavir/ritonavir (n = 140, 41.8% and n = 23, 39.7%). Risk of QT prolongation was present in 232 (55.6%) patients, hydroxychloroquine (n = 159, 68.5%) and lopinavir/ritonavir (n = 70, 30.2%). Overall, 87.4% of patients showed ≥ 1 DDI, recording 1 DDI, 2 DDIs and 3 DDIs in 32.2%, 20.7% and 16.7% of the patients, respectively; a maximum of 10 DDIs were detected in one patient. Overall, there were 60 different medications involved in the detected 417 DDIs.

According to the ATC classification system (Table 2), the majority of the detected DDIs were related to cardiovascular system (38.4%), nervous system (27.6%) and alimentary tract and metabolism drugs (15.3%). Amber DDIs involved mainly furosemide (n = 38, 11.6%), amlodipine (n = 33, 10%) and bisoprolol (n = 28, 8.5%), whereas red DDI involved predominantly the coadministration of metamizole with hydroxychloroquine (n = 13, 22.4%).

**Table 2:** Real DDIs between experimental COVID-19 therapies and comedication according ATC.

First level ATC classification n (%)	ATC classification (second level)	Frequency (%)	
Cardiovascular system (n = 160, 38.4%)	Diuretics (C03)	49	11.8%
	Beta blocking agents (C07)	39	9.3%
	Calcium channel blockers (C08)	33	7.9%
	Cardiac therapy (C01)	14	3.4%
	Lipid modifying agents (C10)	12	2.9%
	Angiotensin system (C09)	11	2.6%
	Antihypertensives (C02)	2	0.5%
Nervous system (n = 115, 27.6%)	Analgesics (N02)	46	11.0%
	Psycholeptics (N05)	33	7.9%
	Psychoanaleptics (N06)	27	6.5%
	Anesthetics (N01)	9	2.2%
Alimentary tract and metabolism (n = 64, 15.3%)	Drugs used in diabetes (A10)	27	6.5%
	Gastrointestinal disorders (A03)	25	6.0%
	Antidiarrheals agents (A07)	7	1.7%
	Antiemetics and antinauseants (A04)	5	1.2%
Systemic hormonal preparations (n = 23, 5.5%)	Corticosteroids for systemic use (H02)	21	5.0%
	Thyroid therapy (H03)	2	0.5%
Antiinfectives for systemic use (n = 19, 4.6%)	Antibacterials for systemic use (J01)	15	3.6%
	Antimicotics for systemic use (J02)	2	0.5%
	Antivirals for systemic use (J05)	2	0.5%
Others		36	8.6%

Regarding ATC, groups more commonly used were: C03-diuretics (11.8%), N02-analgesics (11.0%), and C07-Beta blockers (9.3%) and C08-Calcium antagonists (7.9%).

### Potential DDIs among experimental COVID-19 therapies and the concomitant medications

Overall, 82.8% (n = 144) of participants included in the study had at least 1 comedication. Of those, 63.9% (n = 92) of patients used five and more chronically prescribed drugs. Among the 144 patients with comedication, pDDIs were detected in 105 patients (72.9%), with a total of 553 interactions. Of these pDDIs, 74.3% were amber (n = 411), 16.5% red (n = 91) and 9.2% yellow (n = 51). Lopinavir/ritonavir presented a higher percentage of contraindicated DDIs (n = 45, 49.5%) followed by hydroxychloroquine (n = 31, 34.1%). For amber pDDIs was similar to hydroxychloroquine (n = 187, 45.5%) vs. lopinavir/ritonavir (n = 169, 41.1%). The majority of the detected pDDIs were related to the use of cardiovascular system drugs (45.6%, Table 3).

**Table 3:** Potential DDIs between experimental COVID-19 therapies and comedication according to ATC.

First level ATC classification n (%)	ATC classification (second level)	Frequency (%)	
Cardiovascular system (n = 252, 45.6%)	Diuretics (C03)	77	13.9%
	Beta blocking agents (C07)	73	13.2%
	Calcium channel blockers (C08)	34	6.1%
	Lipid modifying agents (C10)	30	5.4%
	Angiotensin system (C09)	18	3.3%
	Cardiac therapy (C01)	18	3.3%
	Antihypertensives (C02)	2	0.4%
Nervous system (n = 161, 29.1%)	Psychoanaleptics (N06)	79	14.3%
	Psycholeptics (N05)	50	9.0%
	Analgesics (N02)	24	4.3%
	Antiepileptic (N03)	8	1.4%
Alimentary tract and metabolism (n = 64, 11.6%)	Drugs used in diabetes (A10)	57	10.3%
	Gastrointestinal disorders (A03)	7	1.3%
Blood and blood forming organs (n = 57, 10.31%)	Antithrombotic agents (B01)	57	10.3%
Other		19	3.4%

**Figure 1:**
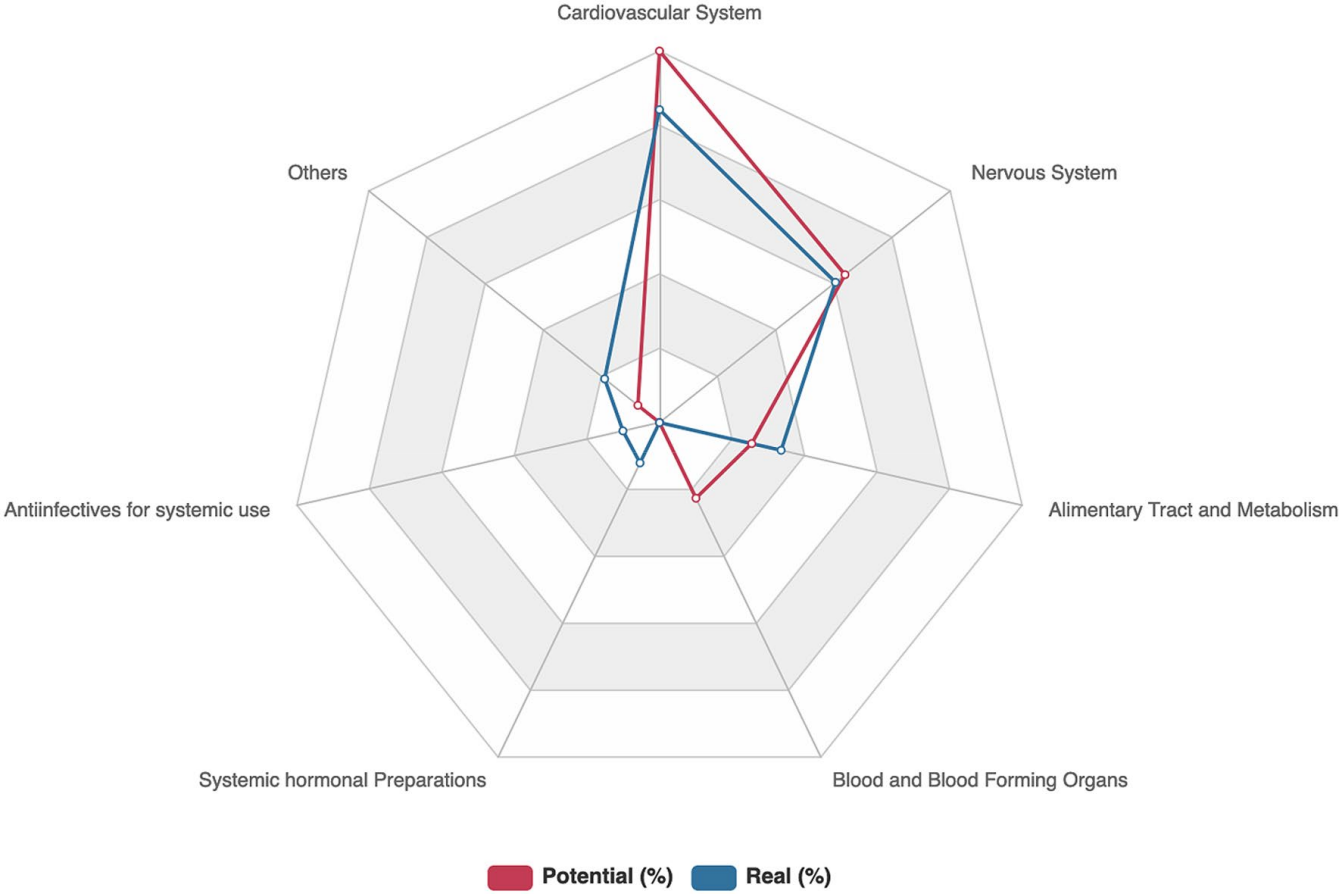
DDIs between experimental COVID-19 therapies and comedication according to ATC classification first level.

Real and potential DDIs comparison between experimental COVID-19 therapies and comedication is shown in Fig. 1.

### Factors associated with real DDIs

**Figure 2:**
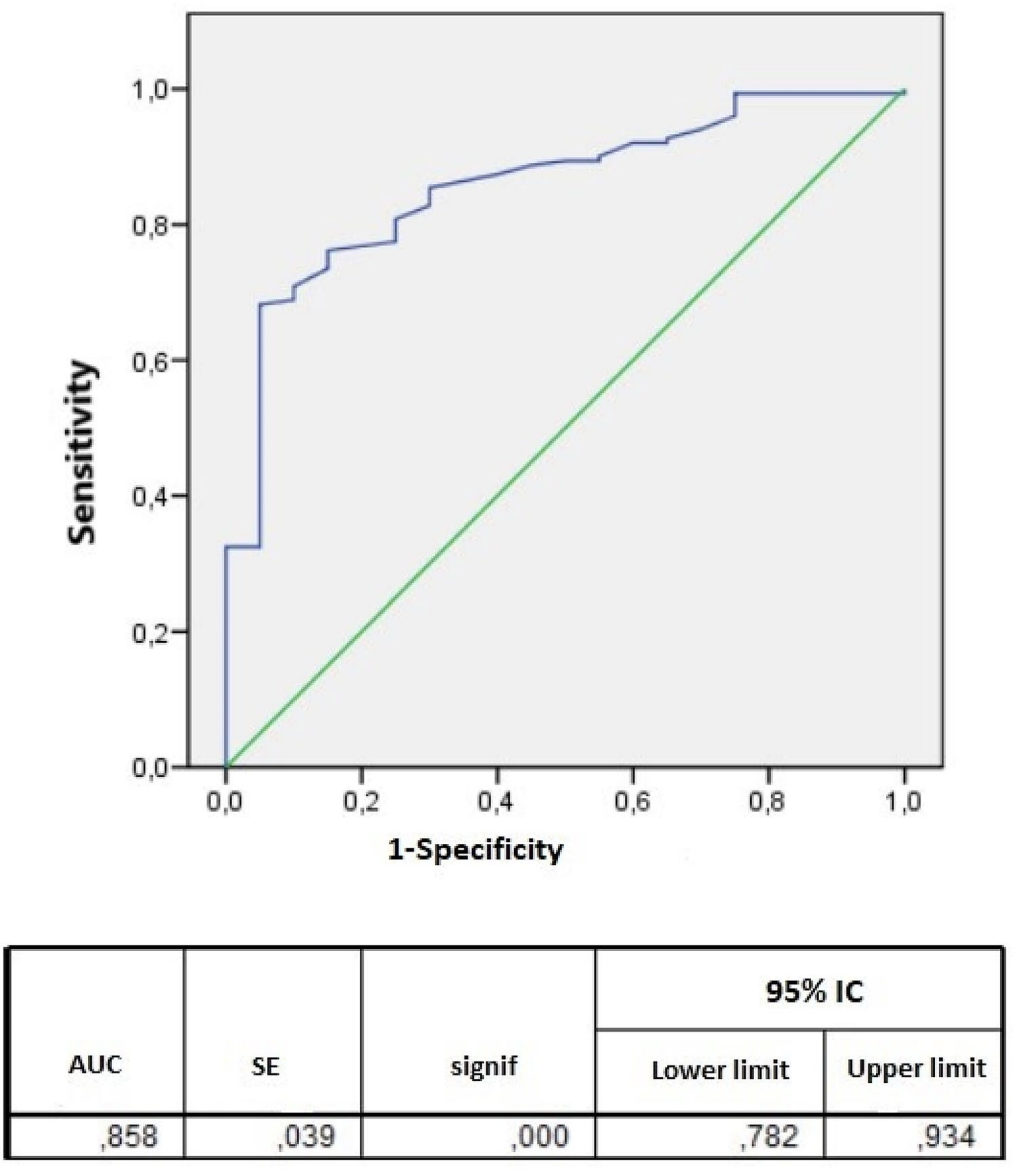
ROC curve for predictive regression model.

We carried out a binomial and stepwise backward logistic regression analysis, based on the wald statistic method to assess independently predictive factors of rDDIs. Higher charlson index (OR 1.34, 95% IC 1.02–1.76) and number of drugs prescribed during admission (OR 1.42, 95% IC 1.12–1.81) were independently associated with the presence of clinically relevant DDIs. In other words, according to our results, for each additional drug prescribed, the occurrence of an interaction is 1.42 times more likely. Additionally, the area under the curve associated with the regression model was graphed, with a result of 0.86 (Fig. 2).

## Discussion

In our study, we identified a high frequency of DDIs between drugs for COVID-19 and concomitant drugs used during admissions. One third of the patients showed multiple 4 or more DDIs. In addition, a high rate of potential DDIs among treatments for COVID-19 and comedications prescribed as outpatients. The results obtained show that patients with COVID-19 have high comorbidity and polypharmacy rates, a circumstance that influences a greater likelihood of real DDIs to therapy for COVID-19.

To our knowledge, this is one of the first studies to address the issue of drug interactions, highlighting the great safety concerns associated with the therapy available at the beginning of the pandemic. Keeping in mind that the age range of the patients most affected in the first wave of the pandemic were those of older age, the use of concomitant medication becomes especially relevant and it is worth highlighting even more the need to evaluate the possible interactions between the experimental medication for COVID-19 and that concomitant medication administered by other pathologies. In our study, the overall comorbidity burden related to patients was elevated. Cardiovascular, metabolic, and mental diseases were the most prevalent. These results are consistent with the literature^3,6,21,22^. We identified a high number of patients showing DDIs. Almost all of them were due to lopinavir/ritonavir and hydroxychloroquine, both treatments are currently in disuse because so far, they have not proven to be useful in the pharmacotherapeutic management of COVID-19^23,24^. Although hydroxychloroquine was the most widely used drug, the greatest number of interactions were associated with lopinavir/ritonavir. Such a high prevalence, 26–47%, is consistent with studies in the HIV population^25,26^. This fact could be explained since ritonavir irreversibly inhibits CYP3A4 and lopinavir/ritonavir induces CYP1A2, CYP2C9, CYP2C19 and glucuronidation. In addition, hydroxychloroquine and lopinavir/ritonavir can cause QTc prolongation, and combined use with other QT-prolonging drugs should be avoided. Moreover, despite we already knew the risk of QT prolongation associated with hydroxychloroquine therapy by itself^27^ as well as combined with azithromycin, it could involve patients diagnosed with COVID-19^28,29^. Although from a clinical point of view it may be understandable that therapies with little evidence are used in times of emergency, it is also important to consider the possible deleterious effect that we can cause if aspects as important as safety are not rigorously evaluated.

The majority of real and potential DDIs reported in this study were clinically relevant. In usual clinical practice these interactions are that require the most attention. The most prevalent contraindicated interaction was simultaneous use of metamizole with hydroxychloroquine. This combination should be avoided due to the increased risk of haematological toxicity. In addition, metamizole is a moderate inducer of CYP3A4 and may decrease hydroxychloroquine concentrations as hydroxychloroquine undergoes CYP-mediated metabolism by CYPs 2C8, 3A4 and 2D6^30^. In this respect, when introducing the experimental drugs, some concomitant drugs could be substituted or dose administered could be reduced. However, the analysis of each interaction is theoretical, and more interaction studies would be needed to confirm its real effect.

The mechanism of interaction involved in most patients is increased the risk of QT prolongation. In a recent study^31^, also in the first pandemic wave, the authors studied potential interactions and reported a prevalence of 62%, slightly lower than our data (72.9%) although probably due to the retrospective design of the study as well as the fact that the adverse effect studied was only the QT elevation. According to the literature, the risk of cardiac arrest was higher in patients receiving more than one QT prolongation drug^20^. This condition is found in most patients since many of them were treated with hydroxychloroquine and lopinavir/ritonavir concurrently, even some patients with concurrent use of five QT prolongation drugs.

In our study, the most common drugs associated with real and potential DDIs were diuretics, analgesics, beta blocking agents, drugs used in diabetes and antithrombotic agents. In concordance, an independent association between real DDIs and Charlson index was found after performing a logistic regression analysis as consequence as between DDIs and comorbidities treated with these drugs (i.e., hypertension, diabetes, ischemic heart disease and heart failure). The association between DDIs and comorbidities as well as polypharmacy has been described in several studies^25,32^. We have observed a higher risk of interactions as more additional drugs are prescribed during admission. In fact, the occurrence of an interaction is 1.42 times more likely for each additional drug. This information is unbelievably valuable, since it allows us to quantify the risk involved in the administration of drugs that to date have not shown benefit against COVID-19, and that can also cause a suspension of other concomitant proven effectiveness-drugs for underlying diseases.

We must recognize several limitations. The retrospective observational design prevents the establishment of causal relationships. In addition, although it is a unicentric study, the characteristics of the population do not differ from the rest of patients who attend other Spanish hospitals^5^. Sample size calculation could be another methodological flaw in the study. However, all COVID-19 patients were analysed during usual clinical practice at a time when information was limited given that the study began at the onset of the pandemic. Another limitation could be that we did not have information regarding the clinical impact of contraindicated drug interaction. To highlight, it was conducted with a high number of patients; this aspect could be considered as strength of the study.

Finally, since in the first wave, in the absence of solid evidence, we have been using drugs that have not shown any usefulness in the treatment of COVID-19, and also ignoring possible harmful interactions and even suspending drugs for underlying diseases that are truly useful (in order to avoid QT elevation, main adverse event known in that moment), from now on it is advisable to be alert to this situation and consider giving more prominence to the hospital pharmacist as an expert in interactions and pharmacovigilance, in order to ensure that patient safety is a priority on the management of this disease in hospital setting. Thus, we believe that it is especially important to monitor these types of interactions in real time as the pandemic evolves and it is therefore essential that health professionals can evaluate where medication benefits can be achieved while avoiding DDIs. Unfortunately, the risk of DDIs is largely unknown since most studies on COVID-19 do not afford information on interaction between drugs used during COVID-19 and co-medications used for the management of other comorbidities.

In conclusion, the prevalence of patients with real and potential DDIs was found to be high. As of these, the most prevalent DDIs were moderate severity. Potentially clinically relevant drug interactions became a major issue during the first wave of the COVID-19 epidemic because prevalence of comorbidities and polypharmacy in patients infected with SARS-CoV-2, as well as using two or more QTc-prolonging drugs. Charlson index and number of drugs prescribed were found as risk factors significantly related to the occurrence of DDIs (Supplementary Information).

## Patients and methods

### Design and study population

An observational single centre cohort study was conducted at a tertiary Hospital in Spain from March 1st to April 30th. All patients with COVID-19 attended at our centre were included in the study if they met the following inclusion criteria: patients age ≥ 18 years with laboratory-confirmed SARS-CoV-2 infection^33^, admitted to a hospitalization ward and on experimental treatment for COVID-19. The study excluded all those patients with a hospital stay < 24 h, or those with no data about pharmacotherapy or demographics. Patients seen at the emergency room department, but not admitted, were excluded. Data collected from the electronic medical records included demographics, clinical and laboratory data. The Charlson comorbidity index (CCI) was used as a general measure of comorbidity and predicted mortality^34^.

DDIs were checked with The University of Liverpool resource according to known pharmacokinetics, cumulative toxicities and QT risk^30^. The severity of them were categorized using four levels: green, as no clinically significant interaction expected; yellow, potential interaction likely of weak intensity, additional action/monitoring, or dosage adjustment unlikely to be required; amber, potential interaction that may require close monitoring, alteration of drug dosage or timing of administration; red, these drugs should not be co-administered (contraindicated). We defined categories “red, contraindicated” and “amber, potential interaction” as the clinically relevant DDIs. Real DDIs refer to interaction with concomitant drugs prescribed during hospital stay whereas potential DDIs (pDDIs) refer to those with domiciliary medication. Finally, medications that were detected to have DDIs were classified according to the Anatomical Therapeutic Chemical (ATC) classification system^35^.

### Statistical analysis

Discrete variables were expressed as counts (percentage) and continuous variables as medians and interquartile ranges (IQRs). Differences in categorical variables were calculated using a two-sided likelihood ratio chi-square test or Fisher exact test, and t-test or the Mann–Whitney U test were used for continuous variables, when appropriate. Logistic regression analysis was carried out to assess factors independently related to the presence of clinically relevant DDIs. The threshold for statistical significance was defined as p < 0.05. Data analysis was performed using SPSS for Windows 21.0 (SPSS, Chicago, IL, USA).

### Ethics approval

The study was designed and performed according to the Helsinki declaration and approved by the Ethics Committee of the Valme University Hospital (Seville, Spain). Informed consent was waived by the Ethics Committee of the Valme University Hospital due to the observational retrospective design of the study.

## Supplementary Information

Supplementary Information.
