# Supplementary material for: Drug–drug interactions between treatment specific pharmacotherapy and concomitant medication in patients with COVID-19 in the first wave in Spain
Source: Sci Rep. 2021 Jun 14;11:12414. doi: 10.1038/s41598-021-91953-2 (PMC8203634; doi:10.1038/s41598-021-91953-2)
Supplement: Supplementary file 1 — Supplementary Information. [file 41598_2021_91953_MOESM1_ESM.docx]

Supplementary Table 1. Logistic regression final model.

|  | **B** | **SE** | **p value** | **OR** | **95%IC** |
| --- | --- | --- | --- | --- | --- |
| **Sex (male)** | 0.013 | 0.558 | 0.981 | 1.013 | 0.34-3.03 |
| **Age** | -0.017 | 0.027 | 0.537 | 0.983 | 0.93-1.08 |
| **Charlson index** | 0.294 | 0.141 | 0.037 | 1.341 | 1.02-1.76 |
| **Number of concomitants drugs** | 0.353 | 0.124 | 0.004 | 1.423 | 1.11-1.81 |
